# Can Music Therapy Improve the Quality of Life of Institutionalized Elderly People?

**DOI:** 10.3390/healthcare10020310

**Published:** 2022-02-06

**Authors:** María José González-Ojea, Sara Domínguez-Lloria, Margarita Pino-Juste

**Affiliations:** 1 Department of Didactics, School Organization and Research Methods, Faculty of Education and Sport Sciences, University of Vigo, 36310 Vigo, Spain; mariajosegonzalezojea@uvigo.es (M.J.G.-O.); mpino@uvigo.es (M.P.-J.); 2 Research Group on Education, Physical Activity, and Health (GIES10), Galicia Sur Research Institute (IIS Galicia Sur), SERGAS-UVIGO, 36312 Vigo, Spain; 3 Department of Special Didactics, Faculty of Education and Sport Sciences, University of Vigo, 36310 Vigo, Spain

**Keywords:** elderly, music therapy, depression, quality of life

## Abstract

Introduction: The current population has new characteristics that require changes to be made in the public health system. In the case of the elderly, the concrete aspects of their health must be known to improve the system, in search of a better quality of life and as much independence as possible. Method: The aim of this study was to verify the efficiency of a music therapy program with institutionalized elderly participants to avoid depressive symptoms and improve social interaction and creativity. This is a group case study that uses a pretest–post-test descriptive design. The program was divided into sixteen sessions, two sessions each week. As inclusion and exclusion criteria, physical dependency and cognitive state were used. Results: The results present an improvement in the physical dimensions of quality of life and an increase in creativity and social interaction. It is recommended that the sessions in the program, aiming to achieve a greater efficiency, are extended because the elderly have very ingrained habits and routines that are very hard to eliminate. Discussion and conclusions: Music therapy, a non-pharmacological and worthwhile treatment, is a therapeutic option with proven benefits. Music therapy has the potential to improve health and quality of life in the elderly and also foster the amelioration of various chronic illnesses, such as depression.

## 1. Introduction

The current population has new characteristics that make transformations to public health oriented towards the aging of the population necessary, so that the system can respond adequately.

It is important to understand health in the elderly to improve it, knowing that it is not only about acting in the health field, but also in everything that influences the environment in which they live [1]. Non-pharmacological interventions improve the quality of life of patients. Non-pharmacological treatments are a very interesting avenue of study in high-income countries.

In the elderly, we do not seek to cure but to improve their quality of life, carrying out activities that positively influence the possibility of them continuing to carry out the basic activities of daily life by themselves. We seek to maintain the physical fitness, functional performance, activity, and quality of life of the elderly through rehabilitation and training programs [2].

Currently, non-pharmacological therapies that help to prevent cognitive decline are increasingly being sought. Music offers great potential for improving well-being in older people and their health [3], with music therapy being a strategy used for the prevention of cognitive decline and in those already suffering from mild cognitive impairment. Beer [4] defends music therapy as a non-pharmacological therapy that is attractive due to its benefits in the treatment of pathologies such as dementia and its symptoms, being a cost-effective type of intervention. 

Quality of life is influenced by individual factors such as the family or residential environment and community factors such as social support, the health system or lifestyle choices [5]. Therefore, in this study, we aim to determine whether a music therapy program can favor social relationships, originality, initiative, or creativity, thus avoiding pathologies such as depression and promoting an adequate quality of life.

## 2. Theoretical Framework

Music is potentially valuable for the improvement of the well-being and health of the elderly. Music therapy and music-related activities foster enjoyment, socialization, well-being, and the improvement of mental health in older people. The elderly are increasingly prone to isolation and suffering from pathologies related to mental health such as, among other things, depression [3].

Music therapy is already established in the United States and is beginning to be used in other developed countries [6]. Every day, more is known about the rehabilitative effect and power of music in older people and in other types of patients [7], so it can be an interesting tool in the field of alternative medicine [8].

Music therapies act through emotional and psychophysiological pathways, reducing anxiety, aggressiveness, improving the mood and autonomy of patients, and helping to alleviate depressive symptoms [9].

Park [10] defends musical activities as part of a strategy with the potential to improve the social well-being of older people, both those who are healthy and those who are institutionalized with cognitive problems. Activities that focus on music, such as playing instruments or listening to different types of music, help to improve the well-being of the elderly, their quality of life, and social health, while helping to reduce their levels of anxiety or depression. 

The WHO [11] defines depression as a “common mental disorder, characterized by the presence of sadness, loss of interest or pleasure, feelings of guilt or lack of self-esteem, sleep or appetite disorders, feeling of tiredness and lack of concentration”. It is a disorder that can become chronic, making it difficult to carry out daily life activities, both at work and at a personal level. In the most serious cases, it can incur a risk of suicide. Depression has negative effects on the quality of life of older people.

Depressive disorders in the elderly are one of the most important and disabling psychiatric disorders in this type of population. They are considered a public health problem as, in developed countries, they are one of the main causes of disability. These pathologies functionally incapacitate people, which prevents them from living independently within a community, maintaining meaningful interpersonal relationships, or achieving personal goals [12].

Regarding the efficacy of music therapy programs in older people, an improvement in depression, memory, orientation, or anxiety was observed from the fourth session of music therapy in older people with a diagnosis of Alzheimer’s [13,14,15]. In India, Dev et al. [16] studied the effects of music therapy in terms of improving depressive symptoms, concluding that it is an effective, safe, and profitable practice. Chu et al. [17] also conclude that group interventions with music therapy consitute a cheap and non-invasive therapy for older people suffering from depression or dementia, since it delays the deterioration of cognitive functions. Music also functions as a rehabilitative tool that improves the social well-being of the elderly and helps to soften depressive symptomatology by lowering anxiety levels [7,8,9,10].

Quality of life is the way in which an individual perceives their life, their place in the cultural context, and the value system in which they live, their objectives, norms, concerns, and criteria, all in relation to their physical health, daily activities, social relationships, psychological state, and degree of independence, environmental factors, and personal beliefs [18].

The use of music therapy improves people’s functionality, which allows for the optimization of quality of life through the early introduction of psychosocial interventions such as the use of music therapy as a non-pharmacological treatment [19].

In people diagnosed with dementia, music therapy improves quality of life by stimulating their psychomotor, cognitive, perceptual, communication and socio-emotional capacities, helping them to be calmer and to work on their emotional expressions and movement [20].

It is, therefore, of interest to carry out research where we can prove the effectiveness of music therapy, not only in the treatment of diseases but also as an alternative therapy for the prevention and improvement of psychological factors that help the elderly to maintain a better quality of life.

Authors such as Särkämö [21] affirm that there is emerging evidence that musical activities have potential benefits for cognitive, emotional, and social functioning, both in normal aging and in different stages of life, with a potentially neuroprotective effect of musical participation for neurodegenerative diseases, with music therapy being a potential tool for improvement and prevention in many of the pathologies of the diseases of aging.

The objective of the present study is to verify the efficacy of a music therapy program with institutionalized elderly people for the consolidation of their quality of life by controlling their anxiety levels and favoring their creativity, and to analyze the modifications that must be made to improve their results.

## 3. Method

The methodological approach used was a group case study with a descriptive and interpretive purpose to be able to explain the actions of the participants considering different sociocultural, temporal, and spatial variables of the scenario where the educational action took place.

A critical approach was used, which makes it possible to provide solutions to specific problems and, thus, improve the social and personal aspects in the situations the program is used on. For this, a group case study has been chosen using a pretest–post-test design. This type of study provides benefits to participants in research processes [22]. The analysis procedures were mixed, as is usual in this type of study [23]. The music therapy sessions were designed based on the objectives of the program and considering the design of Domínguez-Lloria et al. [24] for this type of intervention, using music therapy as a non-pharmacological treatment.

### 3.1. Inclusion and Exclusion Criteria

To decide on whether participants should be included or excluded, their cognitive status, measured with the Pfeiffer scale, and level of dependence in the basic activities of daily living, based on the Barthel scale, were assessed.

The criteria used were a normal or moderate cognitive assessment and a mild to moderate dependence.

### 3.2. Participants

The music therapy program was carried out, with 52 elderly people living in a nursing home taking part. In total, 27.5% of the participants were men and 72.5% women. The mean age of the participants was 83.41 years. 

The choice of the center was probabilistic, since a center was randomly selected from the Baixo Miño region in the municipality of Tui, a city located in the southwest of Spain. The geriatric center selected belongs to a non-profit institution.

A total of 90% of the elderly only have a primary education level, and 50.5% are single and 49.5% are married. Additionally, 56.9% have children, with the average number of children being 1.38. The remaining 43.1% do not have children. The most common profession before retirement was housewife, with a percentage of 29.4%, followed by service sector workers (27.5%), farmers (24.8%), operators (14.7%), professional technicians (3.7%), and, finally, administrative staff, with a percentage of 1.8.

Regarding the clinical context, the residence has a medical team, consisting of a doctor and two nurses, who oversee everything related to health in primary care and manage all consultations and admissions that require specialized care. They also monitor and prevent risk factors in the elderly to try to improve and maintain their state of health. One of these nurses is also a qualified music therapist, which allows for a sustained and planned intervention over time due to the continuous contact with the residents.

The music therapist works with the elderly, mainly on activities for health maintenance, disease prevention, and improvement of quality of life, which arethe main objectives of her interventions. The elderly danced together and the interaction between the music therapist and the elderly was continuous and joint. The music therapist and other professionals were always present during the intervention.

### 3.3. Ethical Considerations

Instruments were administered individually to all residents to assess their baseline health status. All participants voluntarily completed the requested information.

At the end of the program, the post-test was carried out, once again passing the initial assessment scales to the participants. In addition, these data were compared with those collected in the field diary and observational records.

The study was carried out following the deontological standards recognized by the Declaration of Helsinki (Hong Kong revision, September 1989) and in accordance with the recommendations of Good Clinical Practice of the EEC (document 111/3976/88 of July 1990) and current Spanish legal regulations governing research, as well as the CSIC standards published in March 2010 and the agreements of good practices outlined by the Publications Ethics Committee (COPE) [25], as well as the AERA [26] and APA [27] standards.

### 3.4. Instruments

#### 3.4.1. Quantitative Instruments

The Barthel Scale was used to assess physical dependence to carry out the basic activities of daily life. The score on this scale ranges from 0 (total dependence) to 100 (independent). The maximum score drops to 90 in the case of people who are in a wheelchair [28].

The residents’ cognitive status was assessed using the Pfeiffer scale (Short Portable Mental Status Questionnaire). It is a very sensitive and specific scale. It consists of ten items in which the capacity for orientation, memory, concentration, and calculation is evaluated. On this scale, each point is an error in the responses. Between 0 and 2 errors is considered as normal cognitive function, from 3 to 4 errors a mild cognitive impairment, 5 to 7 errors a moderate cognitive impairment, and more than 8 errors a severe cognitive impairment [29].

For the assessment of depression, BDI (Beck’s Depression Inventory) was used, which is the most widely used and researched instrument for measuring depression [30,31,32,33]. It consists of 21 items and the score can vary from 0 to 63 points. Between 0 and 13 points is considered as minimal depression or absence of the disorder, from 14 to 19 mild depression, 20 to 28 moderate depression, and from 29 to 63 points severe depression [34].

#### 3.4.2. Qualitative Instruments

Qualitative instruments were used to monitor the progress of the participants and thus record possible incidents to solve them efficiently and as soon as possible. For this, a field diary and the observational record were used.

The observational record made it possible to monitor the progress seen by the researcher. It was covered in each session individually.

With the use of the field diary, it was possible to record the incidents that occurred during the sessions to act accordingly on them.

### 3.5. Procedure and Data Analysis

Once the participants were selected based on the inclusion and exclusion criteria, the intervention was designed and planned. The music therapist programmed a series of activities focused on rhythm, through choreography and the use of musical instruments (depending on the possibilities of the participants) and singing. 

The program consisted of 16 sessions, 2 sessions a week, starting on 22 February 2020 and ending on 26 April of the same year.

The structure of the sessions was always the same to generate routines and for the participants to familiarize themselves with the activities carried out.

Each of the sessions began with a greeting of approximately 5 min, in which an activity or song chosen by the participants was used, or in which they were reminded of previous sessions.

This was followed by a rhythmic activity, with a duration of 10 min in which, through musical instruments, with clapping and body percussion, rhythmic echoes were first performed to later work on rhythmic patterns that were combined by dividing the participants into two groups and performing a question–answer strategy that was attractive to the participants.

Subsequently, a vocal-auditory activity (15 min) was carried out, in which the aim was to work on memory by singing songs from their childhood, songs that brought back good memories or that were common in their times. 

In the activity related to body expression and psychomotor skills (lasting approximately 10 min), small choreographed routines were performed to try to improve the physical condition and coordination of the participants according to their possibilities. The physical contact and having the possibility to express oneself with the body through music provoked spontaneous physical interaction among the participants.

Finally, a farewell of about 5 min was carried out, where the session ended with a reminder of some of the songs that had been worked on or with one that they proposed to the group, and a dialogue was established about the experience; sometimes, this last activity was replaced by listening to relaxing music.

For the analysis of the data, version 23.0 of the SPSS program was used to record them and perform the analysis using the appropriate statistics for the sample worked. In all cases, a value of *p* < 0.05 was considered significant.

## 4. Results

### 4.1. Depression

If we talk about depressive disorder, it is found that the means are always between 0 and 13, indicating an absence of the disorder (Table 1).

Before the program, there were no significant differences regarding marital status, but some appeared after it was implemented, with a higher score in married people in the somatic-motivational factor.

There is a difference in the children variable: after implementing the program, people with children had a higher score.

In the rest of the variables, there were no significant differences.

In the observational registry data, the depression variable saw little variation during the program. It improved progressively towards the end of the program, but discreetly. This corresponds to the results obtained, since the average was similar before and after the program. The cognitive-affective component had an average of 2.61 before the program and 2.88 after the program. The somatic-emotional component had a mean of 3.67 before the program and 4.11 after the program. The scores are low, which means an absence of depressive disorder.

No differences appeared before or after the program with respect to the educational level variable, which suggests a similarity between the scores.

### 4.2. Quality of Life

Regarding the quality-of-life variable, we can verify that the participants in the program have a medium level of well-being.

In the sex variable, there were differences in physical health, both before and after the program. Men had a higher level of health (xPre = 21.33; xPost = 24.13), and, in both groups, it improved after the program. For the other factors, there are no differences (Table 2).

There are also differences in physical health with respect to the variable marital status, both before and after the program: single people have a higher level of physical health (xPre = 21.26; xPost = 23.56).

Additionally, there are also significant differences both before and after the program with respect to whether they have had children: people that did not have children show a higher level of physical health, (xPre = 21; xPost = 23.22). However, above all, it stands out the difference between those who do have children in the environment variable after the program has been carried out, where the effect size is also average (xPre = 30.88; xPost = 28.79).

However, in the variable level of education, the differences do not occur in physical health but in psychological and environmental health, the quality of life being higher in the participants with higher education levels, also highlighting the size of the effect (psychological health factor xPre = 22.80; xPost = 23.40. Environment factor xPre = 34.80; xPost = 34.20).

Regarding the variables analyzed only through the observational record and the field diary, we find that social interaction improves significantly. At the end of the program, participants can maintain physical contact with each other, although it is less when this contact is between people of different sexes, control the volume of the voice, have greater verbal fluency, attend a conversation, maintain a conversation, ask questions, or respond to questions asked.

Regarding creativity, the aspects valued were fluency (ability to give answers to a problem, solutions or alternatives), speed (time it takes to generate an idea), originality, elaboration, flexibility (ability to change perspective, adapt to new rules or consider a problem from different points of view), redefinition (ability to streamline the mind, free oneself of prejudices or seek different functions than usual), penetration (go beyond the knowledge of each one to see the problems that are not seen by the others) and the connectivity between ideas.

**Figure 1:**
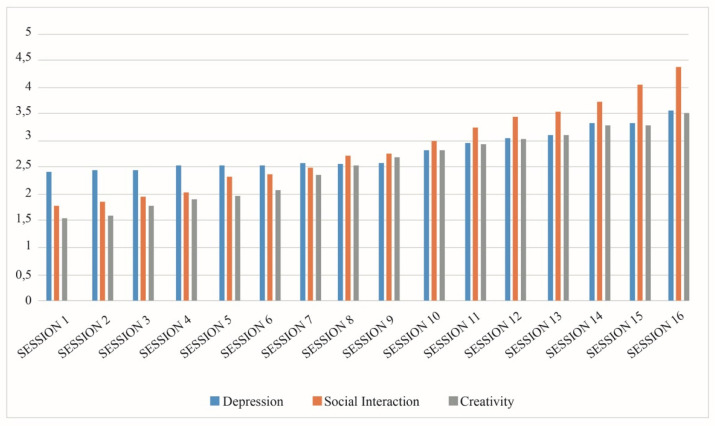
Evolution of depression, social interaction, and creativity of the participants according to the observational record.

Creativity improved differently in different participants. Fluency has been poor, while speed improved a lot as the level of trust between residents increased. Originality and elaboration improved. Flexibility has presented difficulties for almost all participants, since it is difficult for this type of population to consider arguments other than their own. Problems were also found in redefinition and penetration, and in connectivity between ideas (Figure 1).

## 5. Discussion and Conclusions

The purpose of the study was to verify the efficacy of music therapy in improving the health, both physical and psychological, of the elderly.

The studies carried out defend music therapy as a non-pharmacological therapy, without negative side effects and cheap. Thus, various authors defend the value of music to help in the well-being of people, in social relationships, in improving mood, in reducing anxiety and aggressiveness. It is an increasingly studied tool in the field of alternative medicine. Music therapies delay the onset of cognitive decline in older people, as well as helping those who already suffer from it [3,4,5,6,7,8,9,10,11,12,13,14,15,16,17,18,19].

The passage of time and aging lead to the appearance of multiple chronic pathologies, which in turn lead to physical deterioration and the fear of falling down. This fear of falling implies a greater loss of autonomy, a worsening of previous chronic pathologies, and a greater dependence when carrying out the basic activities of daily life, which favors social isolation, cognitive problems, and the appearance of pathologies such as depression [35,36,37].

When people age, changes occur naturally, which, depending on the characteristics of the brain, can lead to cognitive alterations. This onset of deterioration does not start at a fixed age; it is part of normal aging and there is no direct relationship between age and cognitive function [38].

In our study, the program did not obtain marked results at a quantitative level, but it did so at a qualitative level.

Depressive symptoms are based on a feeling of sadness and disability. The environment affects this pathology. The implementation of programs and activities that promote well-being and validity helps to maintain and improve mental health and combat the presence of depressive symptoms.

Music therapy provides beneficial effects with regard to depressive symptoms, helping to reduce anxiety [17,18,19,20,21,22,23,24,25,26,27,28,29,30,31,32,33,34,35,36,37,38,39,40,41,42,43].

The means are similar before and after the program, indicating an absence of depressive disorders before starting. Being aware of age is something important in older people, and it has an impact on greater depressive symptoms [44].

There are no significant differences regarding sex; however, although before the program no differences were detected with respect to marital status, these appeared once the program was implemented. The participants in our study who were married are at this point in their lives either separated or widowed and their level of depression is the same as the participants who are single. In our program, we tried to evoke feelings and positive experiences from their past, which would allow them to remember their good moments as a couple and improve their depression, as argued by Bastida-González et al. [45].

Additionally, people with children have higher scores on the scale. The family environment is an important factor in depression [46]. The presence of family support and having children influence the feeling of loneliness that the elderly can have when they are institutionalized and help to foster a lower rate of depression compared to those elderly who are alone, without family and have a greater sense of abandonment.

Quality of life is defined as the conditions that help the well-being of a person and is directly affected by the environment and the perception of their own well-being, which causes it to decrease depending on the pathologies and problems faced, both physically and psychologically [47].

Well-being is key to the quality of life. Music helps improve well-being, promoting a better quality of life and improving depressive symptoms [3,4,5,6,7,8,9,10].

If we focus on the analysis of the quality-of-life variable, there are no differences in quality of life with respect to the profession variable, but we did detect differences with respect to physical health in the other variables. Men, single people, and those who have not had children score higher in physical health. For the other factors, there are no differences.

In their study, Sánchez Padilla et al. [36] defend the argument that women have worse health, go to the doctor more, and depend on a higher number of drugs than men. This is justified by the roles that women have had throughout history, since they are traditionally caregivers. This explains the reason why men have a better perception of their quality of life in terms of physical health, as well as why it is the single and childless who presented the highest scores, since they have never undertaken the role of caregiver for their family.

In our study, psychological health and environmental health are higher the higher the level of education is; the level of education fosters greater psychological and environmental health, since greater training helps us to face the problems and changes in our environment.

### Limitations of the Study

In our study, we also found some limitations, such as the relatively small sample size caused by the need to use inclusion and exclusion criteria, although the size remained within the usual parameters of this type of study.

There was no control group, so that all residents who so wished, even if they were not within the inclusion group, could participate in the activities carried out. For this reason, it was decided to carry out a pretest–post-test study, with its efficacy evaluated by a comparison of the results.

Another added difficulty was data collection. The physical and cognitive impairments of some of the participants during the intervention made some data unreliable. Even so, it was decided to include them in the study, avoiding altering the results. They were compared with the results of the observational registry and field diary.

The possibility of using a triangulation of perspectives collecting information from other professionals in a direct relationship with the participants in the program and not only with the professionals who carry them out will be valuable in future studies. This would help us to determine more precisely the impact of the program on the variables studied or on others that can be improved through the activities carried out. This impact is often difficult to identify with the observation of a single professional.

Regarding duration, we believe that, given the characteristics of the population studied, it would be necessary to carry out an intervention in phases and evaluate each phase individually. Each of the phases should last approximately four to six weeks. In this way, a feedback mechanism could be implemented when necessary and the optimal time could be determined to verify the changes in the factors to be observed.

The cultural characteristics of the sample population also influenced the measurement instruments. We decided to carry out the surveys individually and as privately as possible, given the suspicion they presented when faced with some questions that they considered out of place, such as those referring to their thoughts about life or sexual health.

## Figures and Tables

**Table 1:** Student’s t calculation for the depression variable before and after the implementation of the program with the independent variables.

	Variable	N	Mean (Pre)	Mean (Post)	SD (Pre)	SD (Post)	T (Pre)	T (Post)	Sig. (Pre)	Sig. (Post)	ES (Pre)	ES (Post)
BDI	Single	23	5.73	5.21	5.585	4.389	−0.660	−2.048	0.513	0.046	−0.091	−0.269
	Marri/Widow	29	6.72	8.41	5.154	6.806						
	Children yes	34	6.38	8.14	4.625	6.481	0.173	1.937	0.863	0.035 *	0.0023	0.0285
	Children no	18	6.11	4.83	6.578	4.449						
	Female	37	6.32	7.51	5.607	6.572	0.076	0.964	0.940	0.339	0.011	0.157
	Male	15	6.20	5.73	4.708	4.333						
	Single	23	2.52	2.21	3.231	2.411	−0.204	−1.513	0.839	0.137	−0.026	−0.214
	Marri/Widow	29	2.68	3.41	2.713	3.122						
	Primary School	47	6.29	7.04	5.421	6.230	0.039	0.155	0.969	878	0.008	0.041
	Secondary School	5	6.20	6.60	4.764	4.037						
	Children yes	34	2.55	3.38	2.451	3.123	−0.190	1.755	0.850	0.085	−0.026	0.262
	Children no	18	2.72	1.94	3.738	2.071						
COGNITIVE	Female	37	2.67	3.10	3.249	3.142	0.231	0.881	0.818	0.383	0.039	0.144
	Male	15	2.46	2.33	1.995	2.023						
	Single	23	3.21	3.00	3.118	2.662	−0.891	−2.053	0.377	0.046 *	−0.124	−0.369
	Marri/Widow	29	4.03	5.00	3.406	4.309						
	Primarios	47	2.68	2.95	3.008	2.918	0.491	0.558	0.625	0.579	0.129	0.137
	Bachiller	5	2.00	2.20	2.121	2.489						
	Children yes	34	3.82	4.76	3.261	4.119	0.452	1.737	0.654	0.088	0.066	0.260
	Children no	18	3.38	2.88	3.380	2.720						
SOMATIC	Female	37	3.64	4.40	3.343	4.139	−0.084	0.868	0.934	0.390	−0.013	0.142
	Male	15	3.73	3.40	3.217	2.667						
	Primarios	47	3.61	4.08	3.172	3.821	−0.375	−0.176	0.709	0.861	−0.075	−0.042
	Bachiller	5	4.20	4.40	4.549	3.714						

* *p* < 0.05. ES = effect size.

**Table 2:** Student’s t calculation of the different factors of quality of life before and after the implementation of the program with the independent variables.

	Variable	N	Mean (Pre)	Mean (Post)	SD (Pre)	SD (Post)	T (Pre)	T (Post)	Sig. (Pre)	Sig. (Post)	ES (Pre)	ES (Post)
PHYSICAL HEALTH	Single	23	21.26	23.56	3.910	4.550	2.984	3.307	0.004 *	0.002 *	0.488	0.516
	Marri/Widow	29	17.68	19.62	4.559	4.039						
	Children yes	34	18.35	20.38	4.702	4.206	−2.138	−2.160	0.039 *	0.036 *	−0.391	−0.292
	Children no	18	21.00	23.22	3.985	5.047						
	Female	37	18.43	20.24	4.173	3.839	−2.127	−2.915	0.038 *	0.005 *	−0.397	−0.480
	Male	15	21.33	24.13	5.108	5.475						
	Primary School	47	18.97	21.04	4.527	4.606	−1.408	−1.549	0.165	0.128	−0.304	−0.342
	Secondary School	5	22.00	24.40	4.949	4.615						
PSYCHOLOGICAL HEALTH	Single	23	20.91	20.86	3.423	2.989	1.199	2.072	0.236	0.043 *	0.167	0.357
	Marri/Widow	29	19.62	18.86	4.169	3.805						
	Children yes	34	19.61	19.05	3.884	3.716	−1.487	−2.105	0.143	0.041 *	−0.213	−0.285
	Children no	18	21.27	21.05	3.722	2.979						
	Female	37	20.16	19.40	4.146	3.685	−0.087	−1.092	0.931	0.280	−0.013	−0.226
	Male	15	20.26	20.60	3.239	3.268						
	Primary School	47	19.91	19.36	19.91	3.522	−3.282	−4.746	0.007 *	0.001 *	−0.535	−0.597
	Secondary School	5	22.80	23.40	22.80	1.516						
ENVIRONMENT	Single	23	31.00	30.73	3.477	3.346	−0.166	1.875	0.869	0.067	−0.022	0.255
	Marri/Widow	29	31.17	28.82	3.910	3.873						
	Children yes	34	30.88	28.79	4.035	3.764	−0.570	−2.439	0.571	0.018 *	−0.087	−0.434
	Children no	18	31.50	31.33	2.995	3.162						
	Female	37	31.40	29.48	3.515	3.761	0.948	−0.561	0.348	0.577	0.138	−0.086
	Male	15	30.33	30.13	4.117	3.777						
	Primary School	47	30.70	29.19	3.568	3.548	−2.477	−3.073	0.017 *	0.003 *	−0.535	−0.743
	Secondary School	5	34.80	34.20	2.863	2.280						
SOCIAL RELATION SHIP	Single	23	8.56	8.69	1.590	1.459	0.325	1.316	0.747	0.194	0.047	0.184
	Marri/Widow	29	8.37	8.03	2.351	2.026						
	Children yes	34	8.61	8.32	2.243	1.995	0.758	−0.018	0.452	0.985	0.115	−0.002
	Children no	18	8.16	8.33	1.581	1.455						
	Female	37	8.78	8.59	1.945	1.640	1.836	1.704	0.072	0.095	0.267	0.240
	Male	15	7.66	7.66	2.093	2.093						
	Primary School	47	8.617	8.46	1.917	1.666	1.723	1.757	0.091	0.085	0.323	0.307
	Secondary School	5	7.00	7.00	2.738	2.738						

* *p* < 0.05. ES = effect size.

## Data Availability

The data are not publicly available due to confidentiality reasons.
